# Using Mobile Apps for Health Management: A New Health Care Mode in China

**DOI:** 10.2196/10299

**Published:** 2019-06-03

**Authors:** Qing Lv, Yutong Jiang, Jun Qi, Yanli Zhang, Xi Zhang, Linkai Fang, Liudan Tu, Mingcan Yang, Zetao Liao, Minjing Zhao, Xinghua Guo, Minli Qiu, Jieruo Gu, Zhiming Lin

**Affiliations:** 1 Department of Rheumatology The Third Affiliated Hospital of Sun Yat-sen University Guangzhou China

**Keywords:** mHealth, internet, health care, medical informatics

## Abstract

**Background:**

China has a large population; however, medical resources are unevenly distributed and extremely limited, and more medical services are needed. With the development and ever-increasing popularity of mobile internet communication, China has created a mode of mobile health (mHealth) care to resolve this problem.

**Objective:**

The aim of this study was (1) to describe the problems associated with China’s medical care practice, (2) explore the need for and the feasibility of internet-based medical care in China, and (3) analyze the functionality of and services offered by internet-based health care platforms for the management of chronic diseases.

**Methods:**

Data search was performed by searching national websites, the popular search engine Baidu, the App Store, and websites of internet medical care institutions, using search terms like “mobile health,” “Internet health,” “mobile medical,” “Internet medical,” “digital medical,” “digital health,” and “online doctor.” A total of 6 mobile apps and websites with the biggest enrollment targeting doctors and end users with chronic diseases in China were selected.

**Results:**

We recognized the limitations of medical and health care providers and unequal distribution of medical resources in China. An mHealth care platform is a novel and efficient way for doctors and patients to follow up and manage chronic diseases. Services offered by these platforms include reservation and payment, medical consultation, medical education assessment, pharmaceutical and medical instruments sales, electronic medical records, and chronic disease management. China’s health policies are now strongly promoting the implementation of mHealth solutions, particularly in response to the increasing burden of chronic diseases and aging in the population.

**Conclusions:**

China's internet-based medical and health care mode can benefit the populace by providing people with high-quality medical resources. This can help other countries and regions with high population density and unevenly distributed medical resources manage their health care concerns.

## Introduction

### Background

China has the largest population and the second largest economy in the world (2018 gross domestic product: 90 trillion Chinese Yuan [CNY]), but medical care resources in China are relatively limited. There are only 2.21 licensed (assistant) physicians for every 1000 people in China. There exists an uneven geographical distribution of medical resources as well. These two problems have led to countless transprovincial patients, resulting in numerous extra economic and time costs.

An understanding of the Chinese health care system and mobile phone usage provides a framework for understanding mobile health (mHealth) apps in China. Acknowledging the increasing pressure exerted by an aging population, behavioral changes, and rapid urbanization [1], the government’s Healthy China 2030 plan [2] envisions the primary health care system as a means of addressing the emerging dual burden of chronic noncommunicable diseases [3-5] and increasing health expenditures. However, primary health care providers in China are usually inadequately trained and some are not even certified [6]. Professionals equipped with up-to-date medical knowledge play an important role in efficient health care, especially among those in poorly developed regions, and the lack of this has become a major issue in China.

With the rapid development of mobile internet communication and the increasing needs of medical services, a new mode of mHealth care is being implemented in China to effectively overcome some traditional barriers. Mobile phones (ie, mobile phones with advanced computing and internet access) and tablet computers have become the most popular and widespread types of mobile devices used. Mobile apps allow patients to access information, assessments, and treatments in a timely manner, and improve the health of those living with chronic diseases [7]. Therefore, extra costs, such those associated with travel, time, and doctor consultations, can be dramatically reduced. In addition, doctors could build another way to connect with their patients, practice without geographical limitation, enhance their reputation by providing remote chronic disease management services to patients, and gain extra income (since they are paid comparatively low wages and minimal benefits due to China’s new health care reform [8]). It can also promote a balanced distribution of medical resources via regional medical information interconnection, which increases the accessibilty of high-quality medical resources in the rural areas. Furthermore, some internet hospitals have been established in China, although many of them are still underdeveloped and face various issues (eg, a scarcity of online doctors) [9]. Many mobile medical platforms, such as Doctor 7LK, Doctor Xingren, Micro-doctor, Doctor Hao, and Doctor Chunyu, usually rely on experts from hospitals. Patients can be reached and followed up via these internet-based platforms. For instance, Doctor 7LK is an comprehensive internet-based medical service enterprise. It can serve as an online practice site for doctors across the country and link hospital resources all over China to provide services such as appointments, remote diagnosis, examinations, and electronic prescriptions to patients.

### Objectives

The aim of this study was (1) to describe the problems associated with China’s medical care practice, (2) explore the need for and the feasibility of internet-based medical care in China, and (3) analyze the functionality of and services offered by internet-based health care platforms for the management of chronic diseases.

## Methods

### Data Collection

Data were collected by searching national websites, the popular search engine Baidu, and websites of internet-based medical care institutions (ie, the National Bureau of Statistics of the People’s Republic of China, the National Health and Family Planning Commission of the People’s Republic of China, the Hospital Management Institute of Fudan University, and the Ministry of Industry and Information Technology of the People’s Republic of China), using search terms like “mobile health,” “Internet health,” “mobile medical,” “Internet medical,” “digital medical,” “digital health,” and “doctor,” until June 2018. In addition, we used Baidu and the App Store to identify medical care platforms and mHealth apps. We found more than 200 medical care platforms and mHealth apps.

### Choosing Medical Care Platforms

We chose the most frequently used mobile medical care platforms, such as Doctor 7LK, Doctor Xingren, Micro-doctor, Doctor Hao, Doctor Chunyu, and Doctor Shu, to analyze the number of registered doctors. Overall, there were 81,604 hospital-based specialists and 400,344 patients involved in these platforms. Subsequently, we compared registered doctors in several provinces on the Doctor 7LK platform, the permanent resident population of these areas, and the distribution of patients and doctors on the Doctor 7LK platform. Data management and analysis were performed using Microsoft Excel 2017. Nominal and ordinal data were presented by using frequencies, percentages, and bar charts.

## Results

### The Overwhelming Health Care Situation in China

**Figure 1:**
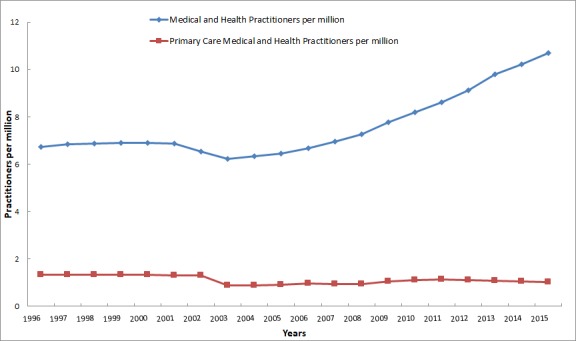
The growth of total medical and health practitioners and primary care medical and health practitioners in the past 20 years in China.

China has limited health care resources and an overwhelming population, but the growth of primary care medical and health practitioners is relatively slow (Figure 1). From 1996 to 2015, the number of health practitioners in China rose by 58.6% from 6.74 million to 10.69 million. In contrast, primary care medical and health practitioners decreased from 1.32 million to 1.03 million, accounting for less than 10% of national medical and health practitioners.

The geographical distribution of medical resources is imbalanced in China. Among the top 100 comprehensive hospitals [1], more than half are located in Beijing (City), Shanghai (City), and Guangdong province, accounting for 23%, 19%, and 9%, respectively. In the 12 western provinces and autonomous regions, only Chongqing, Sichuan, and Shanxi provinces have some of the top 100 hospitals, accounting for 6%, 5%, and 3%, respectively.

### The Current Status of Internet-Based Medical Platforms

In 2018, the coverage rate of mobile broadband network had reached 57%, which is 12 times that of 2002 [10]. By the end of 2016, statistics from the Ministry of Industry and Information Technology of the People’s Republic of China revealed that there were 1.32 billion mobile phone users, 192 times of that observed in late 1996. Furthermore, the mobile phone penetration rate had reached 96.2% [10]. By 2016, the size of China’s mobile medical market had reached 10.56 billion CNY, with a growth rate of 116.4% per year [11].

There are more than 100,000 registered doctors on multiple digital medical platforms [12-17]. For example, the Doctor Hao platform had attracted about 480,000 doctors to register online as of November 2017, followed by Doctor 7LK (389,000 doctors), Doctor Xingren (380,000 doctors), and Micro-doctor (260,000 doctors).

**Figure 2:**
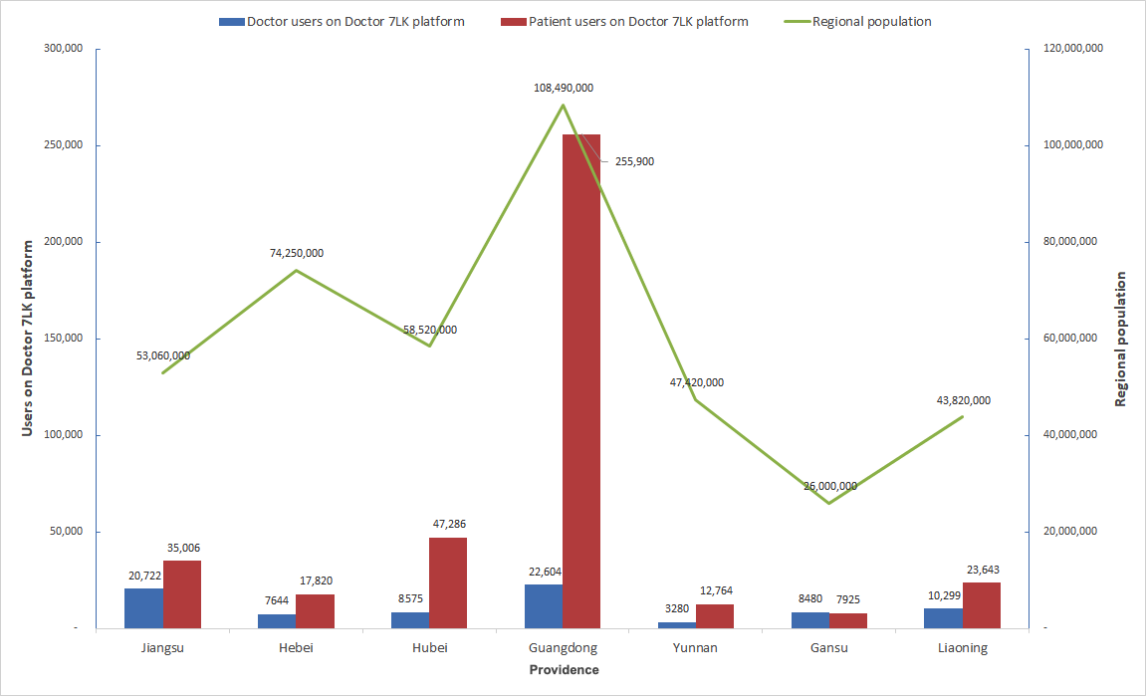
Registered physicians of Doctor 7LK in several provinces and the permanent resident populations of these areas.

The unbalanced distribution of medical resource leads to the exploration and explosion of internet-based medical practice. Although the number of registered doctors in the southeastern area is higher, many doctors are more accepting of an internet-based medical practice in the relatively poor western regions (Figure 2). Gansu province in northwestern China is a good example. The population of Gansu province is 26 million, whereas the registered number of physicians is 8480 on the Doctor 7LK platform, the equivalent of 3.26 online registered doctors per 10 million people. In contrast, 2.08 registered doctors are shared by every 10 million people in Guangdong province, which is much more economically developed and has the largest number of registered doctors. The number of registered patients in the southeastern region is the highest in Guangdong province with 2.36 per 1000 registered patients, far more than that of the central and western regions.

**Figure 3:**
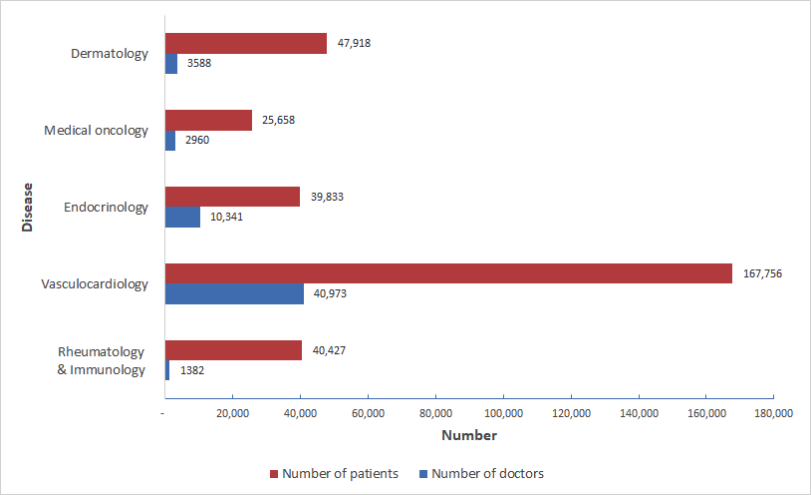
Numbers of patients and the distribution of doctors on the Doctor 7LK platform.

Within the departments of chronic diseases, the largest number of registered users (patients and doctors) came from the cardiovascular division, which is related to the high incidence of cardiovascular diseases in the nation (Figure 3). In terms of the number of patients per registered doctor, rheumatologists were associated with the largest number of patients, with an average of 29.25 patients per rheumatologist, which reflects a relative shortage of rheumatologists at present. Dermatology is the most interactive department and enjoys the largest visit numbers per day, which is linked to special manifestations, diagnosis, and treatment features of skin diseases.

## Discussion

### Principal Findings

We provided an overview of China’s current internet usage and mHealth care mode. The unbalanced distribution of medical resource leads to the exploration and explosion of internet-based medical practice. Internet-based mHealth care platforms could enable people to overcome geographical health care barriers and help reduce the uneven distribution of medical resources. A large number of venture capital funds have flowed into internet-based health care platforms, and the number of users of medical apps has increased gradually each year. Patients in well-developed southeastern China have a higher recognition of internet medical behaviors. Within the departments of chronic diseases, the largest number of registered users (patients and doctors) came from the cardiovascular division, which is related to the high incidence of cardiovascular diseases in the nation.

By 2016, the total population of mainland China had reached 1.38 billion, but there were only 2.21 licensed (assistant) physicians per 1000 people and unlicensed doctors [6]. In the primary health care system, the number of licensed (assistant) doctors per 1000 people in each province ranged from 0.52 to 1.13, and the difference in rural doctors was 16 times (from 0.24 to 3.90). [6]. Similar to the huge differences in the distribution of doctors throughout the entire health care system [18], among the top 100 comprehensive hospitals [19], more than half are located in Beijing, Shanghai, and Guangdong province. On the contrary, the western region of China, which accounts for 72% of the territory and 29% of the whole population, is home to only 14% of the top hospitals. Most of the best resources (doctors) are distributed in large cities such as Beijing, Shanghai, and Guangzhou, although the growth of primary care medical and health practitioners is relatively slow. Due to the huge gap in human resources and clinical skills between general hospitals and primary medical institutions, 3.08 billion patients are accepted by 28,000 general hospitals annually; however, only 4.34 billion patients choose to go to the 921,000 primary medical institutions. This indicates that less than 3% of medical institutions account for more than 40% of medical and health service [2]. One of the main reasons for the shortage of high-quality medical services is that Chinese patients are free to choose their medical institutions and doctors. Even with mild symptoms, there is a tendency to visit high-end hospitals, which results in overcrowding [20].

Why do Chinese patients need the mHealth care platform? First, primary health care providers in China are usually inadequately trained and sometimes uncertified. According to the China Health and Family Planning Statistical Yearbook, only 24% of doctors had a regular license in village clinics in 2015 [6]. The continuing education for primary health care doctors is also insufficient. In addition, primary health care professionals are poorly paid, have the lowest benefits, and the payment policy does not reward high-quality medical services [8]. Skilled doctors are unwilling to work in communities or remote rural areas for financial and professional reasons [21]. In addition, many patients are reluctant to go to primary health care institutions because of a lack of confidence in the health professionals’ skills and the quality of health care provided [22]. The desire to access high-quality care is a trigger for the development of the mHealth care platform.

The good news is that mobile internet communication is booming in China. Statistical data from the National Bureau of Statistics of the People’s Republic of China showed that the broadband network covered 94.5% of the total villages and 78% of impoverished villages by the end of 2015 [1]. The mobile phone penetration rate has been increasing gradually each year. With the development of technology and people’s attention to health, mHealth care platforms, including mHealth apps, have the potential to assist the self-management of many health conditions [7,23] and improve the health of those living with chronic diseases. In the Chinese health app market, the number of technology and health app users is growing exponentially. Although the quality of Chinese mHealth apps appears to be inferior to those in developed countries [24,25], they could help reduce the negative impact of the shortage of professional health care providers and meet the needs for high-quality information and care access [26,27]. We found that a large number of venture capital funds had flowed into internet-based medical platforms, and the number of internet users of medical apps have been growing gradually each year [11]. By the end of 2016, there were over 2000 mobile medical apps in China, involving hundreds of thousands of medical practitioners across the country [12-17].

According to the different stages of medical interventions, operation modes of digital medical services can be divided into different types based on the analysis of these mHealth apps. These include (1) reservation and payment: patients can make appointments with doctors through the platform and pay the consultation fee through the platform; (2) medical consultation and assessment: registered patients can consult doctors on the platform, and the registered doctors can answer the patient’s questions. To ensure the standardization of diagnosis and treatment, these consultations usually involve common medical questions, and the doctors cannot provide diagnostic opinions or prescriptions; (3) medical education: these websites or apps provide patients with disease-related popular science and education articles, such as daily life considerations, self-monitoring, self-management, and emergency response to certain acute diseases; (4) pharmaceutical and medical instrument sales: patients can purchase over-the-counter medications or purchase prescription medicine after verifying a valid prescription on these websites; (5) electronic medical records: the users of such website or apps are usually doctors. It facilitates doctors to make medical electronic records; (6) chronic disease management: such websites or apps usually have doctor and patient versions. These types of platforms usually have multiple functions. For example, doctors can send text messages to patients to remind them about their follow-up visits, and the platforms would automatically make an appointment for a follow-up visit after receiving a reply from the patients. They could also send related medical education articles to the patients under the patients’ request. Doctors can write their patients’ medical records on the platform. Patients can purchase prescription medicine on these websites according to the doctor’s prescription.

Other countries already use mobile phones to assess and treat certain diseases [28-31]. With the widespread availability of the internet and decreasing cost of distant medical services over time, the internet health care mode has had an impact on many developing countries, where medical services and resources are concentrated in large cities. Moreover, the system could improve health education and raise public health awareness, as patients can easily gain access to professional health consultancy.

Chronic disease management platforms aim to facilitate subsequent follow-ups for patients with chronic diseases. It enables remote medical treatment, which otherwise relies on the offline primary medical and health care institutions, clinical laboratories, and medical image centers. The platforms facilitate setting up a medical closed loop of care for doctors, patients, and medicine, thus helping doctors to build up private clinics to better manage patients with chronic diseases. The proportion of doctors and patients associated with chronic diseases is very high; the top 5 departments of registered users are related to chronic diseases. This correlates to the nature of the disease and the need for follow-ups. Using internet-based health care platforms is an innovative and effective way to help doctors follow up and manage their patients with chronic diseases in China.

Nevertheless, in terms of the distribution of registered users, patients in the southeast have a higher recognition of these novel internet-based medical advances. One of the reasons is that it is a relatively economically developed region and the gross regional domestic product is related to higher medical input of the government. Moreover, people in this area are highly educated and they take their health very seriously. However, statistical bias may be caused by the differences among several internet-based medical companies in various areas. The widespread existence of internet-based health care platform has weakened the uneven distribution of medical resources. Many doctors are more likely to accept internet-based medical practices, especially in poor areas as the internet can make it easier for doctors to connect with patients who are geographically distant. Both doctors’ and patients’ needs are fundamental for the survival and development of these platforms.

### Future Prospects

China’s health care system needs to be considerably strengthened to manage both the rising burden of chronic diseases and increasing health-related expenditures [32]. On April 12, 2018, Premier Li Keqiang presided over an executive meeting of the central committee and the state council, which defined measures to accelerate the development of *internet plus medical and health care*, and allowed medical institutions to provide online services and carry out internet-based medical services to benefit more people with high-quality medical resources and improve the overall health of residents. The government will also expand telemedicine coverage to all medical federations, support high-speed broadband networks, and explore the sharing of prescription and drug retail information in medical institutions, thus improving the *internet plus medical care* system [33]. As China takes steps to accelerate the development of *internet plus medical and health care*, it has the opportunity to build an integrated, cooperative internet-based medical and health care system and gain valuable experiences through the whole process. How China‘s internet-based health care will evolve under existing policies and socioeconomic environment is worthy of attention.

### Conclusions

China‘s internet health care mode can provide possible solutions to address the lack and uneven distribution of experienced health care providers in some countries or regions. Although it is still being explored, we highlight the important role of the mHealth care mode in China, which could also be helpful for other developing countries facing similar challenges.

## Abbreviations

CNY: Chinese Yuan
mHealth: mobile health
